# Prognostic value of lymphocyte counts in bronchoalveolar lavage fluid in patients with acute respiratory failure: a retrospective cohort study

**DOI:** 10.1186/s40560-021-00536-w

**Published:** 2021-02-23

**Authors:** Yasutaka Hirasawa, Taka-aki Nakada, Takashi Shimazui, Mitsuhiro Abe, Yuri Isaka, Masashi Sakayori, Kenichi Suzuki, Keiichiro Yoshioka, Takeshi Kawasaki, Jiro Terada, Kenji Tsushima, Koichiro Tatsumi

**Affiliations:** 1 Department of Respirology, Graduate School of Medicine, Chiba University, 1-8-1 Inohana, Chuo-ku, Chiba, 260-8670 Japan; 2 Department of Emergency and Critical Care Medicine, Graduate School of Medicine, Chiba University, 1-8-1 Inohana, Chuo-ku, Chiba, 260-8670 Japan; 3 Department of Pulmonary Medicine, School of Medicine, International University of Health and Welfare, Kozunomori 4-3, Narita, Chiba, 286-8686 Japan

**Keywords:** Bronchoalveolar lavage fluid, Acute respiratory failure, Mortality, Lymphocytes

## Abstract

**Background:**

Cellular patterns in bronchoalveolar lavage fluid (BALF) are used to distinguish or rule out particular diseases in patients with acute respiratory failure (ARF). However, whether BALF cellular patterns can predict mortality or not is unknown. We test the hypothesis that BALF cellular patterns have predictive value for mortality in patients with ARF.

**Methods:**

This was a retrospective single-center observational study conducted in a Japanese University Hospital. Consecutive patients (*n* = 78) with both pulmonary infiltrates and ARF who were examined by bronchoalveolar lavage (BAL) between April 2015 and May 2018 with at least 1 year of follow-up were analyzed. Primary analysis was receiver operating characteristic curve—area under the curve (ROC-AUC) analysis for 1-year mortality.

**Results:**

Among the final sample size of 78 patients, survivors (*n* = 56) had significantly increased lymphocyte and eosinophil counts and decreased neutrophil counts in BALF compared with non-survivors (*n* = 22). Among the fractions, lymphocyte count was the most significantly different (30 [12-50] vs. 7.0 [2.9-13]%, *P* <0.0001). In the ROC curve analysis of the association of BALF lymphocytes with 1-year mortality, the AUC was 0.787 (*P* <0.0001, cut-off value [Youden index] 19.0%). Furthermore, ≥20% BALF lymphocytes were significantly associated with increased survival with adjustment for baseline imbalances (1-year adjusted hazard ratio, 0.0929; 95% confidence interval, 0.0147–0.323, *P* <0.0001; 90-day *P* =0.0012). Increased survival was significantly associated with ≥20% BALF lymphocytes in both interstitial lung disease (ILD) and non-ILD subgroups (*P* =0.0052 and *P* =0.0033, respectively). In secondary outcome analysis, patients with ≥20% BALF lymphocytes had significantly increased ventilator-free days, which represents less respiratory dysfunction than those with <20% BALF lymphocytes.

**Conclusions:**

In the patients with ARF, ≥20% lymphocytes in BALF was associated with significantly less ventilatory support, lower mortality at both 90-day and 1-year follow-ups.

## Background

Acute respiratory failure (ARF) is a common and life-threatening medical condition that is conventionally defined by an arterial oxygen tension of <60 mmHg or as a triad of clinical signs, radiographic findings, and gas exchange alternations [1]. Nearly 2 million people are hospitalized with ARF annually in the USA, and the mortality of ARF remains over 20% [2]. Etiologies of ARF include pneumonia, interstitial lung disease (ILD), acute respiratory distress syndrome (ARDS), malignant tumors, and congestive heart failure. The broad spectrum of the etiologies requires comprehensive diagnostic investigation. Bronchoalveolar lavage (BAL) fluid (BALF) using fiber-optic bronchoscopy can help diagnose a wide variety of lung diseases [3–5]. Following diagnosis, prediction of clinical outcomes of ARF is key for clinical management since this prediction may contribute to the improvement of clinical outcomes.

Substantial studies have revealed the diagnostic value of BALF cellular patterns in patients with lung diseases, including acute and chronic bilateral infiltrative lung disease [6, 7]. However, investigation of the value of BALF cellular patterns for prediction of mortality remains insufficient. Previous studies have investigated the prognostic value of BALF cellular patterns in patients with chronic respiratory failure, including those with hypersensitivity pneumonitis (HP) [8] and idiopathic pulmonary fibrosis (IPF) [9–11]; however, investigation of the prognostic value of BALF cellular patterns in patients with ARF is sparse.

Herein, we test the hypothesis that BALF cellular patterns have prognostic value in patients with ARF. The primary measure was 1-year mortality as a long-term outcome. Short-term (90-day) mortality and ventilator-free days were analyzed as secondary outcomes.

## Methods

### Ethical approval

All study procedures involving human participants were approved by the Human Ethics Committee of the Graduate School of Medicine, Chiba University (no. 2584). This study was designed and conducted in accordance with the ethical principles of the 1964 Helsinki Declaration and subsequent amendments. The requirement for informed consent was waived by the ethics committee because this retrospective analysis was limited to preexisting data collected as standard-of-care by respiratory physicians. Data anonymization was used, and privacy issues were protected. Approval for the opt-out consent method was given by the Chiba University Hospital.

### Study design and definition

This single-center retrospective study included consecutive patients who had ARF and underwent BAL between April 2015 and May 2018 at Chiba University Hospital, Japan. ARF was defined by the following criteria: worsening or development of dyspnea manifested within 30 days, newly emerging bilateral ground glass opacities, and/or the consolidation on chest computed tomography scan, PaO_2_/FiO_2_ ratio of less than 300 mmHg in patients with or without oxygen therapy or non-invasive ventilation (NIV), or, regardless of the PaO_2_/FiO_2_ ratio, patients with mechanical ventilation. Patients with hypoxia caused by heart failure, fluid overload, or pulmonary thromboembolism were excluded.

The etiologies of ARF, including infection, collagen vascular disease, malignancy, or others, were categorized as ILD or non-ILD. ILD included organizing pneumonia (OP), HP, eosinophilic pneumonia, IPF, acute interstitial pneumonia, drug-induced lung injury, and non-specific interstitial pneumonia. HP was diagnosed based on the American Thoracic Society (ATS)/Japanese Respiratory Society (JRS)/Asociación Latinoamericana de Tórax HP clinical practice guideline of 2020 [12]. IPF was diagnosed based on the ATS/European Respiratory Society/JRS/Latin American Thoracic Association IPF guidelines of 2018 [13]. Etiologies were diagnosed based on microbiological evaluations, BALF cellular patterns, and radiographic and pathologic findings by experienced respiratory physicians.

### BAL procedure and BALF analysis

Experienced respiratory physicians performed BAL using a fiber-optic bronchoscope in compliance with ATS guidelines [6] and the institutional criteria. A total of 150 mL of sterile saline solution were divided into three 50-mL aliquots and instilled into the most affected lobe. After instillation of each aliquot, the solution was gently recovered, and the BALF was filtered through sterile gauze and centrifuged to remove mucus and cells. The total cell number was counted manually using a hemocytometer. Differential cell counts were conducted after centrifugation (1 min, 1500 rpm) and Wright–Giemsa staining.

### Statistical analysis

The primary outcome was 1-year mortality. The primary analysis included the generation of an area under the receiver operating characteristic curve (ROC-AUC) to evaluate the prognostic value of BALF for 1-year mortality. The optimal cut-off value was calculated using the Youden index. The secondary analysis included a Cox regression to test significant effects on hazard of death over 1-year. We chose this approach to adjust for baseline imbalances in variables, including PaO_2_/FiO_2_ ratio and ILD, based on previous reports [14]. The Cox regression analysis was repeated in ILD and non-ILD subgroups. The secondary outcome variables were 90-day mortality and days alive and free of ventilator out of 28 days (VFD). Data are expressed as median and interquartile range. Baseline characteristics were analyzed using Fisher’s exact test or Mann–Whitney *U* test. Two-tailed *p* values <0.05 were considered as significant. All analyses were performed using JMP pro 13.2.0 (SAS Institute Inc. Cary, NC, USA).

## Results

**Fig. 1:**
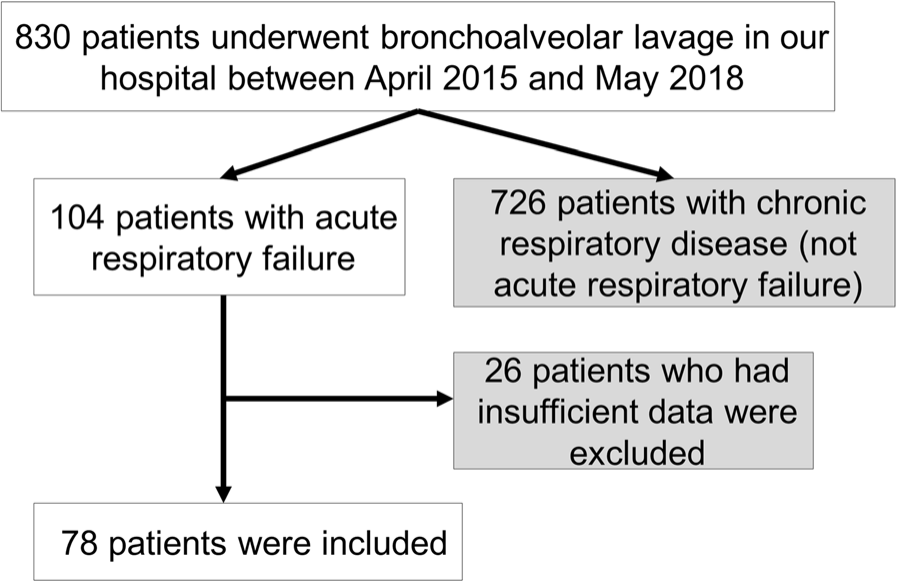
Study flow chart. Of the 830 patients who underwent bronchoalveolar lavage during the study period, we excluded 752, leaving a final sample size of 78

There were 830 patients who underwent BAL during the study period. Of these, 78 who had ARF were analyzed (Fig. 1). At baseline (Table 1), non-survivors had a significantly lower prevalence of ILD and a significantly higher prevalence of requirement for NIV or mechanical ventilation at the time of BAL compared to survivors. Lymphocyte and eosinophil counts were significantly decreased, and neutrophil counts were significantly increased in the BALF of non-survivors compared to that of survivors (Table 1).

**Table 1:** Characteristics and outcomes between survivors and non-survivors at 1 year

	Survivor (*n* = 56)	Non-survivor (*n* = 22)	*P* value
Baseline characteristics			
Age (years)	68 (59–76)	72 (56–76)	0.40
Male, no. (%)	40 (71)	17 (77)	0.60
Body mass index (kg/m^2^)	22 (19–26)	21 (20–24)	0.61
Comorbidity, no.			0.97
Infection/Malignancy/CVD	4/10/6	1/4/1	
HT, HL, DM/others/non	19/14/3	8/7/1	
Diagnosis, no. (%)			
Interstitial lung disease	32 (57)	6 (27)	0.024
Non- interstitial lung disease	25 (42)	18 (75)	
Infection/malignancy/CVD/others, no.	12/4/6/2	5/2/3/6	
Laboratory data on day of BAL			
White blood cell count (10^3^/μL)	8.8 (6.2–11)	11 (5.9–14)	0.15
Neutrophils (%)	82 (72–91)	84 (79–91)	0.16
C-reactive protein (mg/dL)	7.4 (2.3–17)	10.6 (6.9–18)	0.20
Lactate (mg/dL)	1.1 (0.9–1.5)	1.6 (1.0–2.3)	0.11
PaO_2_/FiO_2_ ratio	233 (183–279)	201 (164–254)	0.14
BAL findings			
MV or NIV at BAL, no. (%)	13 (23)	12 (55)	0.014
Recovered BAL fluid (%)	51 (30–61)	43 (29–53)	0.14
Total cell counts in BALF (10^4^/mL)	28 (17–46)	30 (10–44)	0.58
Fractionation in BALF (%)			
Macrophages	38 (20–57)	37 (16–60)	0.87
Neutrophils	12 (3.5–38)	53 (12–72)	0.0016
Lymphocytes	30 (12–50)	7.0 (2.9–13)	<0.0001
Eosinophils	1.0 (0–2.0)	0 (0–2.1)	0.022
Treatments			
Methylprednisolone pulse, no. (%)	32 (57)	13 (59)	1.00
Corticosteroids therapy, no. (%)	45 (80)	17 (77)	0.76
Initial dose of corticosteroids (mg/kg)	0.80 (0.46–1.0)	0.98 (0.79–1.14)	0.23
Outcome			
Length of hospital stay (days)	30 (19–53)	39 (18–96)	0.28

*BAL* Bronchoalveolar lavage, *BALF* Bronchoalveolar lavage fluid, *DM* Diabetes milieus, *CVD* Collagen vascular disease, *HT* Hypertension, *HL* Hyperlipidemia, *MV* Mechanical ventilation, *NIV* Non-invasive ventilation

Data are presented as median and interquartile range for continuous variables. *P* values were calculated using Pearson’s chi-squared test, Fisher’s exact test, or the Mann–Whitney *U* test

**Fig. 2:**
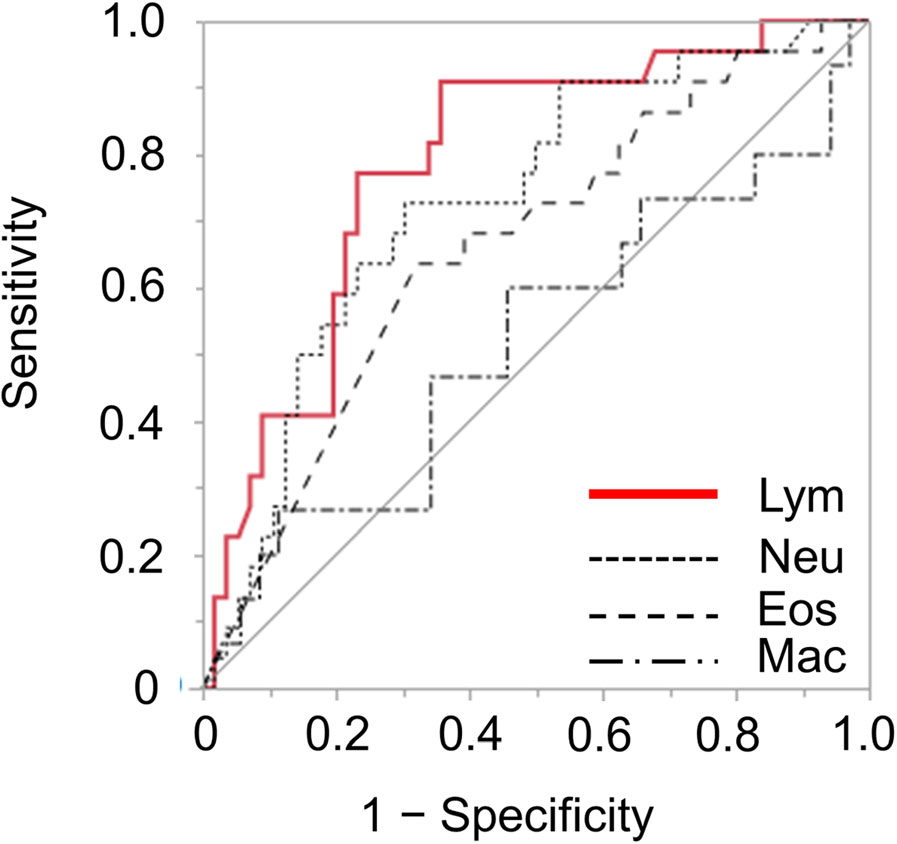
Receiver operating characteristic curves for prediction of 1-year in-hospital mortality. Lym, lymphocytes; Neu, neutrophils; Eos, eosinophils; Mac, macrophages

In the primary analysis of the prognostic value of BALF for 1-year mortality using AUC analysis, lymphocytes had the highest prognostic value among four fractions (AUC, 0.787; 95% confidence interval [CI], 0.657–0.878; *P* <0.0001). The cut-off value for the lymphocyte fraction in predicting 1-year mortality was 19.0% (sensitivity, 0.909; specificity, 0.643) (Table 2, Fig. 2). With respect to the Youden index findings, we defined 20% as the cut-off value for lymphocytes in BALF in 1-year mortality (sensitivity, 0.909; specificity, 0.607) for clinical implication.

**Table 2:** Receiver operating characteristic curve analysis for prediction of 1-year mortality using each factor at differentiation of BALF

Variable	AUC (95% CI)	*P* value	Cut-off value	Sensitivity, specificity	OR (95% CI)
Lymphocytes	0.787 (0.657–0.878)	<0.0001	19.0	0.909, 0.643	0.940 (0.904–0.978)
Neutrophils	0.731 (0.593–0.836)	0.0018	28.3	0.727, 0.696	1.027 (1.009–1.045)
Eosinophils	0.662 (0.525–0.776)	0.047	0.0	0.636, 0.679	0.879 (0.713–0.999)
Macrophages	0.521 (0.336–0.700)	0.91	80.8	0.182, 0.982	1.001 (0.981–1.022)

*BALF* Bronchoalveolar lavage fluid, *CI* Confidence interval, *AUC* Area under the curve, *OR* Odds ratio

Cut-off value was calculated using Youden index

**Fig. 3:**
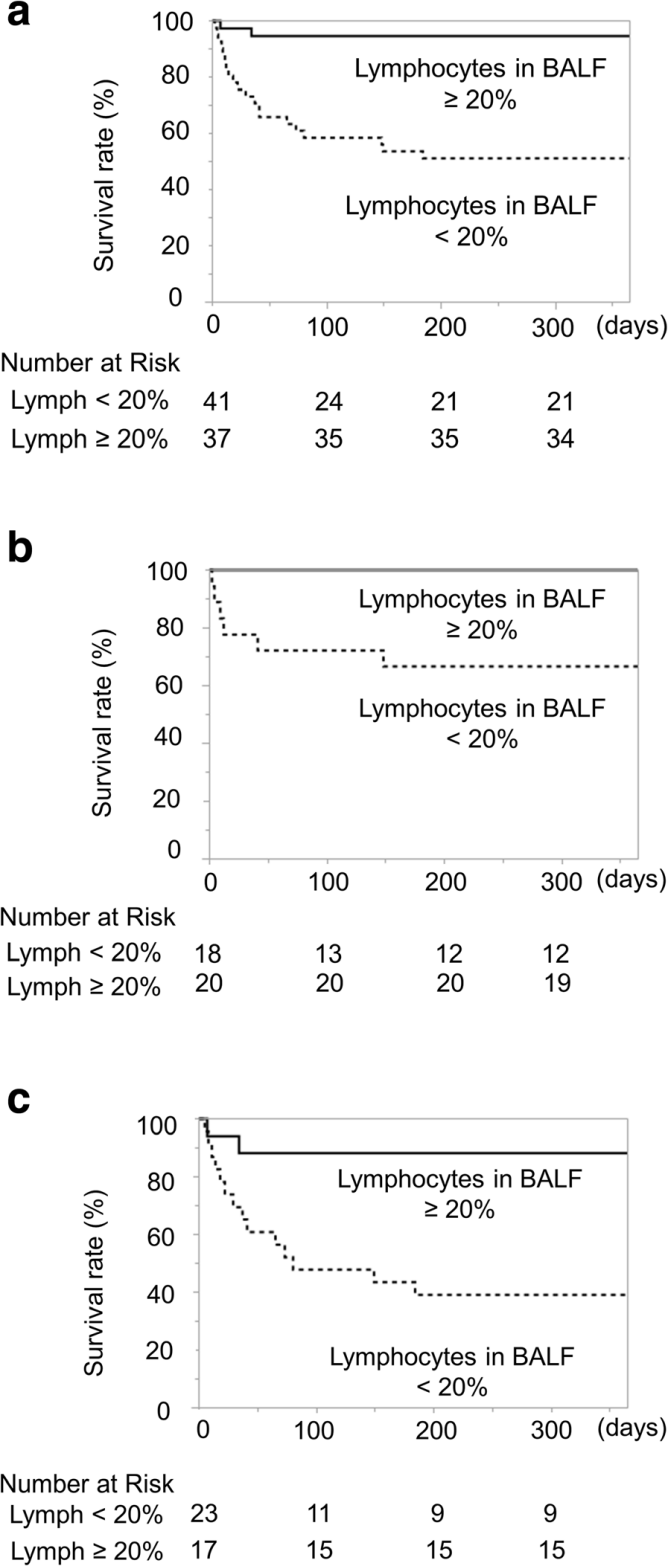
Survival curves in patients with acute respiratory failure (ARF) parsed by lymphocytes in bronchoalveolar lavage fluid. **a** All patients, *P* < 0.0001. **b** Interstitial lung disease, *P* = 0.0052. **c** Non-interstitial lung disease, *P* = 0.0033. *P* values were calculated using the log-rank test

In the secondary analysis using Cox regression, we categorized the patients into those with ≥20% BALF lymphocytes and those with <20% BALF lymphocytes based on significant findings from the primary analysis. The number of survivors and non-survivors with ≥20% lymphocytes in BALF at 1 year was 35 and 2, respectively. The number of those with <20% lymphocytes in BALF at 1 year was 21 and 20, respectively. Survival rates at 1 year were 94.6% in ≥20% BALF lymphocytes and 51.2% in <20% BALF lymphocytes, respectively (Fig. 3a). Patients with ≥20% BALF lymphocytes had significantly decreased mortality after adjusting for baseline imbalances (adjusted hazard ratio, 0.0929; 95% CI, 0.0147–0.323; *P* <0.0001) (Table 3). Repeat analysis of 90-day mortality yielded the same conclusion (Table 3). Subgroup analysis of ILD and non-ILD groups also revealed significantly decreased mortality in the ≥20% BALF lymphocyte subgroup. In ILD, survival rates were 100% in ≥20% BALF lymphocytes and 66.7% in <20% BALF lymphocytes, respectively (Fig. 3b). In non-ILD, survival rates at 1 year were 88.2% in ≥20% BALF lymphocytes and 39.1% in <20% BALF lymphocytes, respectively (Fig. 3c).

**Table 3:** Association between bronchoalveolar lavage fluid lymphocytes ≥20% and decreased mortality

	Hazard ratio (95% CI)	*P* value
1-year mortality		
P/F ratio	0.996 (0.990–1.00)	0.18
Interstitial lung disease	0.375 (0.133–0.926)	0.033
BALF lymphocytes ≥20%	0.0929 (0.0147–0.323)	<0.0001
90-day mortality		
P/F ratio	0.997 (0.991–1.00)	0.27
Interstitial lung disease	0.371 (0.120–0.964)	0.041
BALF lymphocytes ≥20%	0.177 (0.041–0.534)	0.0012

*BALF* Bronchoalveolar lavage fluid, *CI* Confidence interval, *P/F* PaO_2_/FiO_2_

*P* values were calculated using a multivariate logistic regression analysis

Next, we analyzed the association between ≥20% BALF lymphocytes and VFD. A BALF lymphocyte fraction of ≥20% was significantly associated with increased VFD compared to <20% BALF lymphocytes (13 [1-28] vs. 28 [28-28] days, *P* <0.0001), which indicates that an increased BALF lymphocyte fraction is associated with better respiratory outcomes.

## Discussion

In the study of BALF in patients with ARF, the BALF lymphocyte fraction had significant prognostic value. Patients with ≥20% BALF lymphocytes had significantly increased survival at both the 90-day and 1-year observational time points and significantly less ventilator support during the 28-day period.

To our knowledge, the prognostic value of BALF lymphocytes in survival has not been documented in ARF. A key finding of the present study is that BALF lymphocytes had high prognostic value for predicting 1-year mortality in ARF patients (AUC, 0.787; cut-off value, 19.0%). Specifically, patients with ≥20% BALF lymphocytes had significantly decreased 1-year mortality compared to patients with <20% BALF lymphocytes. In line with this finding, a previous investigation of acute exacerbations of chronic progressive interstitial pneumonia with a small sample size (*n* = 37) showed that patients with ≥15% BALF lymphocytes had significantly decreased mortality compared to patients with <15% BALF lymphocytes [14]. Since repeat analysis of 90-day mortality revealed the same results, we concluded that BALF lymphocytes have high prognostic value and that ≥20% BALF lymphocytes are a potential threshold in ARF.

A BALF lymphocyte fraction of >15% represents a lymphocytic cellular pattern in ILD including OP, drug-induced pneumonitis, HP, and non-specific interstitial pneumonia [6]. Indeed, a previous report defined OP and drug-induced pneumonitis that were identified using open-lung biopsy as corticosteroid-sensitive pathologies in non-resolving ARDS [15]. These corticosteroid-sensitive pathologies were associated with better outcome. OP is one of the most common pathologies in ILD and is caused by idiopathic etiologies or is secondary to an inflammatory reaction to drugs, infection, collagen vascular disease, malignancy, or radiation therapy.

The mechanism of increasing lymphocytes in BALF remains unclear; however, natural killer and natural killer T-like cells, which are unique subgroups of lymphocytes, are increased in BALF of OP patients and may play important roles in disease [16]. They also mainly mediate innate anti-tumor and anti-viral immune responses and respond to a variety of cytokines [17]. Therefore, we speculated that some immune responses to tumor, infectious pathogens, and autoantibodies may also affect disease behavior such as secondary OP and improve survival rate for corticosteroid sensitivity in both ILD and non-ILD patients with ARF.

The present analysis of ventilator-free days revealed that patients with ≥20% BALF lymphocytes had less ventilatory support. VFD is a standard indicator of respiratory failure including that in patients with ARDS [18–20]. Indeed, improvement of respiratory physiology and shortened ventilator duration is clinically and economically meaningful [21]. Therefore, our findings strengthen primary analysis showing that mortality is linked to respiratory dysfunction.

Finally, it should be noted that our study was limited by being conducted retrospectively. Some patients with ILD were diagnosed without pathological findings because it was difficult to perform surgical lung biopsy in critically ill patients. In most patients who were excluded, recovering an adequate amount of BALF for analysis was challenging because their respiratory condition was unstable. Further large-scale studies must be conducted to confirm the findings of the current study.

## Conclusions

In conclusion, BALF lymphocytes had significant prognostic value for 1-year survival in patients with ARF. Patients with ≥20% BALF lymphocytes had less ventilator support and significantly higher 1-year survival.

## Data Availability

The datasets used and/or analyzed in the current study are available from the corresponding author upon reasonable request.

## Abbreviations

ARDS: Acute respiratory distress syndrome
ARF: Acute respiratory failure
ATS: American thoracic society
AUC: Area under the receiver operating characteristic curve
BAL: Bronchoalveolar lavage
BALF: Bronchoalveolar lavage fluid
HP: Hypersensitivity pneumonitis
ILD: Interstitial lung disease
IPF: Idiopathic pulmonary fibrosis
JRS: Japanese respiratory society
NIV: Non-invasive ventilation
OP: Organizing pneumonia
ROC: Receiver operating characteristic
ROC-AUC: Receiver operating characteristic curve—area under the curve
VFD: Days alive and free of ventilator out of 28 days
